# Why Don't You Try Harder? An Investigation of Effort Production in Major Depression

**DOI:** 10.1371/journal.pone.0023178

**Published:** 2011-08-10

**Authors:** Marie-Laure Cléry-Melin, Liane Schmidt, Gilles Lafargue, Nicolas Baup, Philippe Fossati, Mathias Pessiglione

**Affiliations:** 1 Motivation, Brain and Behavior lab (MBB), Institut du Cerveau et de la Moelle épinière (ICM), Hôpital de la Pitié-Salpêtrière, Paris, France; 2 Institut National de la Santé et de la Recherche Médicale (INSERM UMRS 975), Centre National de la Recherche Scientifique (CNRS UMR 7225), Université Pierre et Marie Curie (UPMC-Paris 6), Paris, France; 3 CNRS UMR 8160, Université Lille Nord-de-France (Lille 3), Villeneuve d'Asq, France; 4 Service de psychiatrie adulte, Hôpital Bicêtre, Assistance Publique – Hôpitaux de Paris (AP-HP), Le Kremlin-Bicêtre, France; 5 Centre Emotion, CNRS USR 3246, Hôpital de la Pitié-Salpêtrière, Paris, France; Federal University of Rio de Janeiro, Brazil

## Abstract

Depression is mainly characterized as an emotional disorder, associated with reduced approach behavior. It remains unclear whether the difficulty in energising behavior relates to abnormal emotional states or to a flattened response to potential rewards, as suggested by several neuroimaging studies. Here, we aimed to demonstrate a specific incentive motivation deficit in major depression, independent of patients' emotional state. We employed a behavioral paradigm designed to measure physical effort in response to both emotional modulation and incentive motivation. Patients did exert more effort following emotionally arousing pictures (whether positive or negative) but not for higher monetary incentives, contrary to healthy controls. These results show that emotional and motivational sources of effort production are dissociable in pathological conditions. In addition, patients' ratings of perceived effort increased for high incentives, whereas controls' ratings were decreased. Thus, depressed patients objectively behave as if they do not want to gain larger rewards, but subjectively feel that they try harder. We suggest that incentive motivation impairment is a core deficit of major depression, which may render everyday tasks abnormally effortful for patients.

## Introduction

The affective experience of depressed patients is characterized by exacerbated negative feelings (fatigue, sadness, guilt, self-depreciation) and blunted positive feelings (energy, pleasure, hope, confidence). These constructs are targeted by numerous questionnaires that are common clinical tools used to assess depressive symptoms, such as the Montgomery and Asberg Depression Rating Scale (MADRS) [1] or the Hospital Anxiety and Depression scale (HAD) [2]. Cognitive neuroscience has extensively investigated the negative aspects of depression, bringing evidence for excessive processing of negative stimuli and memories [3], [4], generally associated with hyperactivity of the amygdala, anterior insula or anterior cingulate cortex [5], [6], [7], [8], [9]. More recently, the blunting of positive emotions have come under scrutiny [10], [11], with several studies reporting a diminution of pleasurable experiences (anhedonia), often associated with reduced activation of the striatum and orbitofrontal cortex [12], [13], [14], [15], [16]. These studies suggest that anhedonia may relate to the loss of motivational drive commonly observed in these patients.

Despite the wealth of questionnaire and neuroimaging data, the difficulty experienced by depressed patients in energising behavior remains poorly understood. In particular, it is unclear whether their emotional state prevents patients from taking action, or if they suffer from a primary motivation deficit. Motivation can be defined as a set of processes that translate goal representation into behavioral activation [17], [18]. A goal is what we call an expected reward, i.e. an anticipated enjoyable experience (e.g. earning money, winning awards or being loved) for which we would spend more energy (e.g. working harder, planning strategies or learning new tricks). Incentive motivation consists of suggesting a goal to initiate a behavior, for example, we might try to cheer up a depressed friend by suggesting going to a movie. An intuitive prediction is that incentive motivation would fail to activate goal-directed behaviors in depressed patients. To test this prediction, we need some tool to measure behavioral activation, i.e. the amount of energy expended. Indeed, classical measures such as hit rates and response times failed to detect any diminution of incentive sensitivity in major depression [15].

Our team has developed paradigms using a handgrip force measurement device to assess how much effort participants would exert in various situations [19], [20]. In a recent functional neuroimaging study [21], we have dissociated the effects of emotional arousal (induced by incidental pictures) and incentive motivation (induced by monetary rewards). At the behavioral level, both arousal and incentive factors resulted in more effort, with a much larger effect of monetary incentives in healthy subjects. At the neural level, different pathways were found to drive motor areas: arousal effects involved the ventrolateral prefrontal cortex and incentive effects involved the limbic basal ganglia. In the present study, we used the same behavioral paradigm to assess the integrity of the emotional and motivational pathways leading to physical effort exertion in patients with major depression. The first aim of this study was to test for a specific motivation deficit: we hypothesized that depressed patients would not increase their effort for higher incentives but would remain sensitive to our emotional manipulation. We have also shown in the above-mentioned study [21] that healthy people feel a same objective effort as easier if they are more aroused or motivated. The second aim of the present study was to test whether alleviation of effort sensation by potential rewards is lost in major depression. It would mean that, contrary to the common impression that depressed patients make no effort to improve their lot, they are already close to the maximum they can withstand, even when doing seemingly easy work. To this second aim we asked patients to rate their effort (how hard did you try?) after every trial.

Thus, we administered to depressed patients and healthy controls a behavioral paradigm that manipulates two factors (emotional state with pictures and incentive motivation with money) and records two variables (objective grip force and subjective effort rating). We then used the dependent variables to show that emotional and motivational factors of effort production can be dissociated in depressed patients. Finally we assessed whether these variables could provide reliable markers of major depression.

## Methods

### Participants

The study was approved by the Pitié-Salpêtrière Hospital ethics committee and was supported by the INSERM (the French National Institute of Health). All patients gave written informed consent prior to participation and all data were recorded anonymously. Participants were informed that monetary earnings in the task would be fictive. This was implemented to avoid discrimination, following on the ethics committee recommendations: indeed, it would be unfair to penalize patients financially for their deficits. Note that money was equally fictive for controls and patients, and that previous studies obtained similar results with virtual and real money [20], [21], [22]. It may be argued that the motivation triggered by virtual money is more related to performance scoring than to financial interests.

A total of 22 patients (aged 43.3±2.9 years, 5/17 males/females) were recruited from the Hôpital de la Pitié-Salpêtrière, Clinique du Château de Garches and Maison de Santé de Bellevue. All 22 patients fulfilled the DSM-IV criteria for a non-psychotic major depressive episode (excluding bipolar disorders). Diagnoses were made using the Mini International Neuropsychiatric Interview (MINI) [23]. Exclusion criteria were: under 18 years of age, psychotic manifestation, psychiatric comorbidity (severe personality or anxiety disorder), neurological illness, other medical condition susceptible to affect cognition, current and/or past diagnosis of substance (drug and alcohol) abuse and administration of electro-convulsive therapy in the preceding 12 months. A majority of patients had suffered from at least 3 previous depressive episodes with hospitalization and therapy. All patients were hospitalized and tested within 10 days following the start of antidepressant treatment. Note that many patients had other ongoing treatments, such as anxiolytics and mood stabilizers. More clinical details are provided in Table 1.

**Table 1:** Clinical data.

	Depressed patients (N = 22)
**MADRS score**	31.8±1.0
**HAD anxiety score**	14.0±0.9
**HAD depression score**	13.4±0.8
**Apathy (Starkstein) score**	20.9±1.3
**Disease duration (years)**	5.6±1.5
**Duration of index episode (months)**	3.3±0.6
**Number of depressive episodes**	2.7±0.5
**Number of hospitalisations for depression ± SEM**	2.4±0.4
**Duration of antidepressant (days) ± SEM**	5.4±0.7

Data are means ± inter-subjects standard errors. MADRS  =  Montgomery and Asberg Depression Rating Scale [1]; HAD  =  Hospital Anxiety and Depression scale [2]. Starkstein's scale is a questionnaire that was designed to detect apathy (score > 14) in Parkinson's disease [39].

A total of 26 healthy controls (aged 44.8±2.8, 9/17 males/females) were recruited from the community. They were interviewed with the MINI and were excluded if there was any evidence of psychiatric or neurological illness, psychoactive substance abuse or dependence, or current medication that might have influenced cognitive skills. There was no significant difference between patients and controls in terms of age, gender and education level (see Table 2).

**Table 2:** Demographic and behavioral data.

	Patients (N = 22)	Intra *P*-value	Controls (N = 26)	Intra *P*-value	Inter *P*-value
**Gender (F/M)**	17/5	-	17/9	-	0.37
**Age (years)**	43.3±2.9	-	44.8±2.8	-	0.69
**Education (years)**	6.3±0.2	-	6.7±0.1	-	0.07
**Arousal rating (1-9)**	5.6±0.2	-	4.8±0.3	-	**0.030**
**Maximal force (Newtons)**	249.2±19.2	-	320.5±19.6	-	**0.013**
**Raw effort rating (0-10)**	5.6±0.2	-	5.6±0.2	-	0.62
**Mean grip force (%)**	46.8±2.9	-	45.0±2.3	-	0.62
**Incentive effects**					
**- on grip force (%)**	0.4±1.0	0.70	17.1±3.7	**<0.001**	**<0.001**
**- on effort rating (%)**	13.0±3.5	**<0.01**	-12.8±4.9	**0.015**	**<0.001**
**Arousal effects**					
**- on grip force (%)**	3.5±0.9	**<0.001**	0.0±0.7	0.96	**0.002**
**- on effort rating (%)**	14.9±12.6	0.25	1.5±2.4	0.54	0.26

Significant t-tests (*P*<0.05) appear in BOLD. Intra-group tests are comparisons with null effects. Inter-group tests are comparisons between depressed patients and healthy controls. Data are given as means ± inter-subjects standard errors. Behavioral data are expressed as percentages of the highest measure. Incentive effects are calculated as the difference between 1€ and 0.01€ trials. Arousal effects correspond to the difference between emotional (pooling positive and negative) and neutral pictures.

### Data acquisition

**Figure 1:**
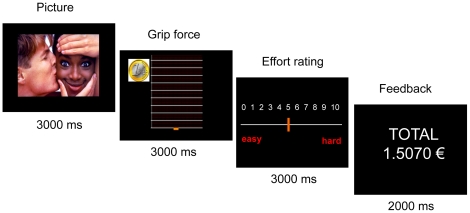
Behavioral task. Successive screenshots displayed in one trial are shown from left to right, with durations in ms. Neutral or arousing pictures (with positive or negative valence) were shown prior to physical effort exertion. Effort was cued by simultaneously showing the amount of money at stake, materialized as coin images (1 cent, 10 cents or 1 euro), and a graduated scale in which a cursor represented the force exerted on the handgrip. Subjects knew that the top of the scale corresponded to the monetary incentive, such that the more they squeezed the handgrip, the more money they would win. After force production, subjects rated the extent of their effort by positioning a cursor on an analog scale. The final screen informed subjects about the cumulative total of monetary earnings.

Stimulus presentation was programmed on a PC using Paradigmae software (Paradigmae, e(ye)BRAIN, Paris, France, www.eye-brain.com). Force was recorded using a hand grip (MIE medical research ltd., Leeds, UK) with a sample rate of 25 Hz. The PC screen provided subjects with real-time visual feedback on the force being exerted on the grip, which appeared as a cursor moving up and down within a grid (see Fig. 1).

In the incentive force task, subjects squeezed a handgrip to win as much money as possible. They were encouraged to perform as if they were playing for real money. The maximal force was measured before starting the experiment: subjects had three trials to squeeze the grip as hard as they could. The average of these three trials was retained to calibrate the scale that served to give subjects visual feedback on the force produced. This was implemented so that the different subjects played for similar amounts of money. After receiving instructions, they were trained on a short practice version (9 trials) in order to become familiarized with stimulus presentation and handgrip manipulation. The task itself was made up of 12 repetitions of 9 trial types, for a total of 108 trials, grouped in a single session lasting about 20 minutes. The trial types were generated according to 3 emotional categories (negative, neutral, and positive) and to 3 monetary incentives (0.01, 0.1, and 1€). Emotional categories and monetary incentives were randomly distributed over the trials and the sequence was fixed such that patients and controls were assessed on the exact same task.

In every trial (Fig. 1), subjects first watched a new emotional picture displayed on screen for 3000 ms. They were told that the content of these pictures would not influence the amount of money they could win. A graduated scale then appeared on screen, together with a coin image indicating the amount of money at stake. This was the cue for subjects to squeeze the handgrip so as to move the cursor up as high as possible, within a 3000 ms interval. Subjects were aware that the height they reached within the scale determined the fraction of the monetary stake they would keep. On the subsequent screen, subjects were asked to rate the effort exerted when squeezing the handgrip. The question was “how hard did you try?” and subjects were encouraged to report their feeling and not to rely on visual feedback (cursor height). To avoid the latter, we randomly varied the height the cursor could rise to (60, 70 or 80% of the scale) when subjects produced their maximal force. To indicate their rating, subjects had to move a cursor within a scale graduated from 0 (“easy”: minimal effort) to 10 (“hard”: maximal effort). They used the keyboard to move the cursor right and left and had 3000 ms to reach the appropriate position. At the end of the trial, feedback for the cumulative amount won so far was presented for 2000 ms.

Emotional pictures were chosen based on valence and arousal ratings provided by the International Affective Picture system [24], [25]. Categories were derived from the valence ratings, with ranges of 1.51–3.94 for negative, 4.30–5.88 for neutral, and 6.29–8.34 for positive pictures. We controlled for luminance and dimension between pictures, and balanced the proportion of social and non-social pictures between categories. As a further control, we asked subjects to rate the arousal and valence of the pictures used in the incentive force task. This rating task was administered after the force task and lasted about 20 minutes on average. Each of the 108 pictures was displayed again for 3000 ms, and subjects had to choose a figure first on the 1–9 arousal scale (1  =  minimal arousal, 9  =  maximal arousal), then on the 1–9 valence scale (1  =  maximal negative valence, 5  =  neutral valence, 9  =  maximal positive valence). To indicate the chosen figure, subjects pressed the corresponding button on the keypad. There was no time limit to respond: subjects moved on to the next picture at their own pace by pressing the button. Two depressed patients could not complete the rating task.

### Data analysis

Several dependent variables were considered: objective grip force, subjective ratings of perceived effort, valence and arousal ratings of emotional pictures. To analyze grip force, we extracted in every trial both the maximum reached and the area under the curve over the 0-6s period following onset of the coin image. We retained only the latter -which takes into account how long in addition to how hard subjects tried- in the below results section, as the two measures gave very similar results. Grip force was expressed as a percentage of the highest measure recorded during task completion, in order to eliminate individual differences in maximal power. Effort ratings were divided by the actual force produced, on a trial-by-trial basis, in order to get an index of subjective effort sensation for a same objective force of 1 Newton. The incentive effect was defined as the difference between 1€ and 0.01€ and the arousal effect as the difference between emotional (averaging positive and negative) and neutral pictures.

We examined whether these effects could accurately classify individuals into controls and patients, using Receiver-Operating-Characteristic (ROC) curves. For each cut-off level on the considered effect (for instance, each size of incentive effect on grip force), we plotted true positive against false positive rate. The best criterion was defined as the cut-off level giving the point closest to 100% of true positives and 0% of false positives. We hereafter report sensitivity (true positive rate) and specificity (100 minus false positive rate) of classifications obtained with the best possible criterion. We also tested correlations between observed effects and clinical scores, in our sample of depressed patients (n = 22). Note that in such a small sample, correlations lack statistical power and have only exploratory value.

A global 3-way analysis of variance (ANOVA) was first conducted to test main effects of group (patients and controls), emotion (positive, negative or neutral) and motivation (0.01, 0.1 or 1€), as well as interactions. When appropriate, post-hoc comparisons were then performed using paired t-tests. Because we found no significant effect of valence, in line with our previous study [21], we pooled the data obtained with positive and negative pictures for subsequent statistical analyses. Multiple linear inter-trial regressions were performed to assess the relative weight of emotional and motivational factors on force production. Z-scored incentive levels and both arousal and valence ratings were included as explanatory variables, with grip force as the variable to be explained. Regression coefficients were estimated at the individual level and then tested for statistical significance at the group level using paired t-tests. Three statistical significance thresholds were considered: *P*<0.05, *P*<0.01, *P*<0.001. All statistical tests were conducted with the Matlab Statistical toolbox (Matlab R2006b, the Math Works, Inc., USA).

## Results

We first examined control measures: ratings of emotional pictures and measures of maximal force. There was no significant difference in valence ratings, but arousal ratings were higher in depressed patients compared to healthy controls for positive and negative (not neutral) pictures (5.6±0.2 vs 4.8±0.3, t_46_ = 2.24, *P*<0.05 when pooling positive and negative categories). Patients thus expressed a greater emotional sensitivity for both positive and negative pictures, with no valence effect. The absolute maximal force (in Newtons) was lower in patients compared to controls (249.2±19.2 vs 320.5±19.6 N, t_46_ = 2.57, *P*<0.05), for both genders (males: 369.2±42.0 vs 418.8±22.4 N; females: 213.9±12.4 vs 268.5±17.3 N). In the incentive force task, this difference was compensated for by scaling the visual feedback to the maximal force for each subject (see methods). Using a relative measure (proportion of maximal force) allowed patients to play for the same range of rewards as controls, but the results may give the impression that patients globally deployed similar amount of force. It must be kept in mind that absolute forces (in Newtons) were lower in patients relative to controls.

**Figure 2:**
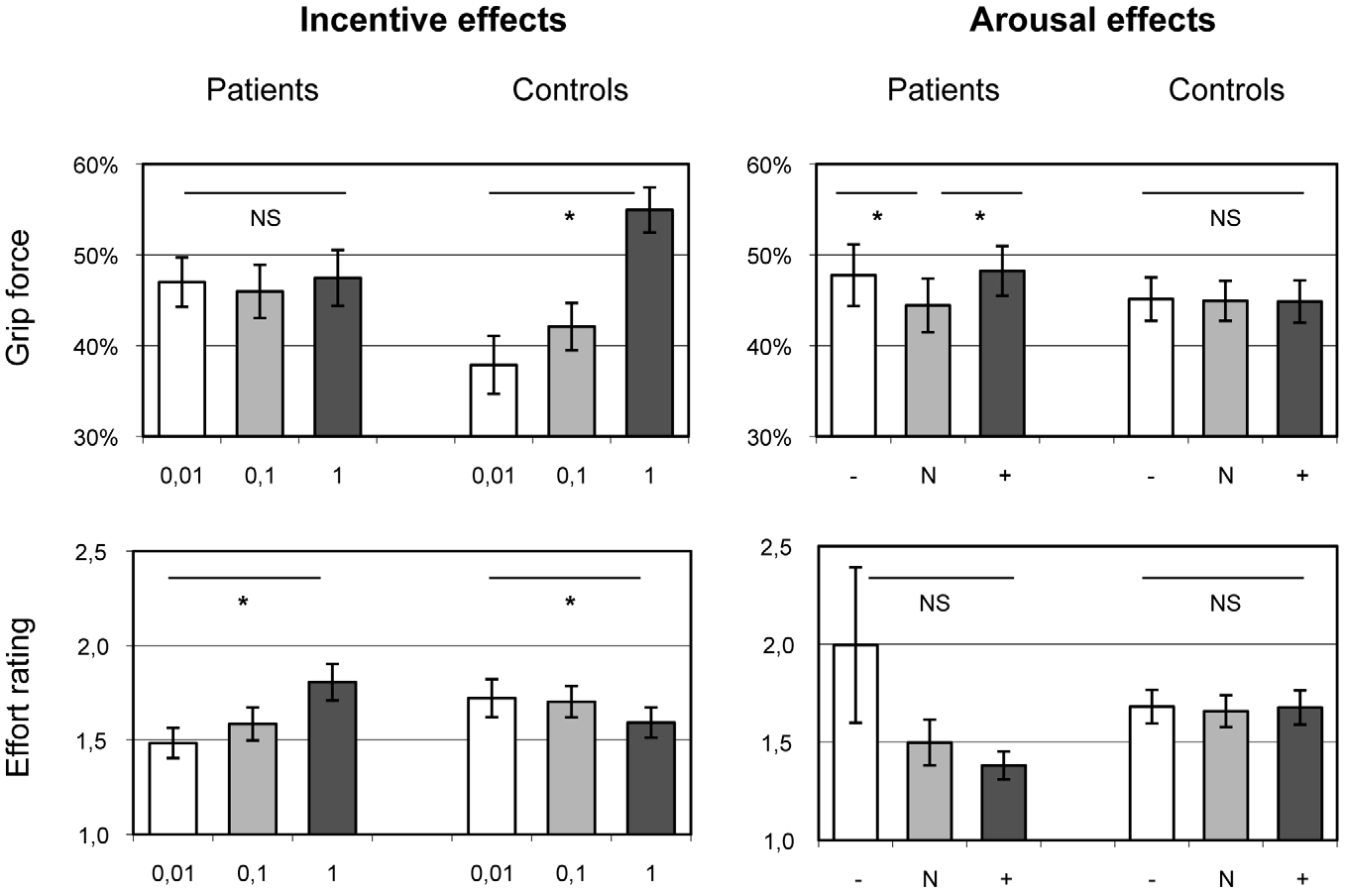
Group-level results. Histograms show the effects of the main independent factors (incentive and arousal levels) on the main dependent variables (grip force, effort rating). Grip force is expressed as a percentage of the highest measure. Effort ratings were divided by the actual force produced on a trial-by-trial basis. Error bars are ± inter-subjects standard errors of the mean. * Significant difference (paired t-test, *P*<0.05), between negative and neutral picture and between 0.01€ and 1€ in the task. NS = non significant.

We then analyzed data for the incentive force task, starting with our main dependent measure, handgrip force (Fig. 2). The global 3-way ANOVA on grip force data recorded in the 48 subjects (22 patients and 26 controls) showed no effect of group, no effect of emotion, and a significant effect of motivation (F_2,414_ = 14.43, *P*<0.001). The only significant interaction was between group and motivation (F_2,414_ = 12.16, *P*<0.001); there was no interaction between emotion and group, or emotion and motivation, and no triple interaction. Thus, the only significant between group difference from this global analysis was the effect of incentive levels on force production. Post-hoc tests indicated that the significant interaction between group and motivation was due to a lower incentive effect in patients compared to controls (0.4±1.0 vs 17.1±3.7%, t_46_ = 4.03, *P*<0.001). Specifically, the incentive motivation effect was not different from zero in patients (0.4±1.0%, t_21_ = 0.38, *P*>0.5), whereas it was significantly positive in controls (17.1±3.7%, t_25_ = 4.60, *P*<0.001). Thus, results provide evidence for incentive motivation deficit in major depression, as potential rewards failed to energize force production.

As picture valence had no significant difference for either group (see Fig. 2), we pooled positive and negative conditions in the following analyses, to assess the effects of emotional arousal relative to neutral state. Emotional arousal effects were greater in patients than in controls (3.5±0.9 vs 0.0±0.7%, t_46_ = 3.27, *P*<0.01) and significant in patients only (3.5±0.9%, t_21_ = 4.15, *P*<0.001), not in controls (0.0±0.7%, t_25_ = 0.06, *P*>0.5). Thus, emotional arousal is a factor that enhances force production in depressed patients, whether the valence is positive or negative. This effect on force production was in fact driven by social pictures (social: 5.4±1.1%, t_21_ = 5.33, *P*<0.001; non-social: 1.8±1.1%, t_21_ = 1.61, *P*>0.1). The null result in controls seems to contradict a previous report of effort facilitation by emotional arousal [21]. However, the latter study only included young subjects (age lower than 39, with an average of 24 years). When we restricted the analysis to the younger half of subjects (age below 45 years), we found that emotional arousal enhanced force production to a similar extent (1.4±0.8%) as in the previous study (1.9±0.4%), although the effect was not significant due to reduced number of subjects. In fact, arousal effects tended to decrease with age in both patients and controls, as shown by Pearson's correlation coefficients (r = −0.26, n = 22 and r = −0.25, n = 26). The same was true for incentive effects: they tended to diminish with age in both groups (r = −0.28, n = 22 and r = −0.37, n = 26).

We also performed regression analyses across trials to assess the relative contribution of emotional arousal and incentive motivation to force production, in both groups. Note that, as in our previous study, there was no interaction between arousal and incentive levels, which were independently manipulated in our experimental design. Regression coefficients were significantly higher for arousal ratings in patients (0.11±0.04 vs 0.02±0.03, t_21_ = 2.24, *P*<0.05) and for incentive levels in controls (0.34±0.29 vs0.03±0.03, t_25_ = 4.41, *P*<0.001). Thus, regression analyses confirmed that grip force was specifically affected by arousing pictures in patients, and by incentive levels in controls. Regression coefficients obtained for valence ratings were not significant, neither in patients nor in controls (r = 0.02±0.02 and 0.02±0.03), supporting the previous finding that positive and negative pictures had a similar effect on force production.

Next we examined effort ratings, normalized according to the force produced on a trial-by-trial basis. As with grip force, the only significant result with the global ANOVA was the interaction between group and motivation (F_2,414_ = 3.21, *P*<0.05). There was a significant difference in incentive effects between patients and controls (13.0±3.5 vs -12.8±4.9, t_46_ = 4.13, *P*<0.001). This difference was due to higher incentives increasing effort sensation in patients (13.0±3.5, t_21_ = 3.70, *P*<0.01), but decreasing it in controls (-12.8±4.9, t_25_ = 2.60, *P*<0.05). There was no significant difference in arousal effects, although in patients there was a non-significant trend for higher effort ratings following arousing pictures (14.9±12.6 vs 1.5±2.4, t_46_ = 1.13, *P*>0.1). This trend was mainly due to marginal patients occasionally reporting higher effort sensation following pictures with negative valence (see Fig. 2).

**Figure 3:**
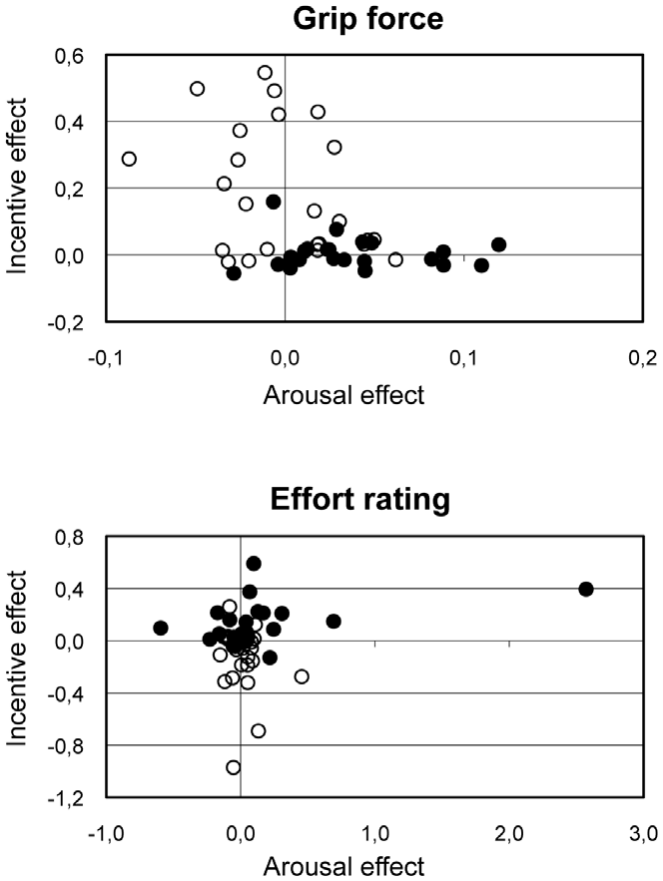
Individual results. Each point is a healthy subject (empty circle) or a depressed patient (filled circle). Graphs show difference scores for incentive (1€ - 0.01€) versus arousal (emotional - neutral) effects on force production and effort rating. Grip force and skin conductance is expressed as a percentage of the highest measure. Effort ratings were divided by the actual force produced on a trial-by-trial basis.

We further investigated whether arousal and incentive effects on our experimental variables could discriminate the two populations (controls and patients). For the two variables (grip force and effort rating), the best discrimination was obtained with incentive effects (Fig. 3). The more sensitive variable was grip force, which shifted distributions of individuals towards higher arousal effect for patients, and higher incentive effect for controls. There was still an overlap however, suggesting that, although it revealed strong differences between groups, our paradigm would not be sufficiently discriminative to be used for individual diagnosis. Indeed, considering incentive effects on grip force, the best discrimination criterion derived from the ROC curve indicates 64% sensitivity and 88% specificity.

Finally, we assessed whether the main result (the absence of incentive effects) was correlated with clinical features. All patients were tested during the first days of antidepressant medication, presumably before any therapeutic effect. However, 9 patients were under other medications (notably antipsychotics) in addition to antidepressants. We found no difference in incentive effects between the 13 patients on antidepressants alone and the 9 patients taking other medications (0.2±1.6 vs 0.7±1.0, t_20_ = 0.24, *P*>0.5). Thus, it seems that medications did not interfere with our main effects, either because it was too early (for antidepressants) or because incentive motivation was already impaired (for antipsychotics). Also, there was no significant correlation between incentive effects and clinical scores of apathy, anxiety and depression (r = 0.32, r = −0.09, r = 0.01 respectively, all *P*>0.5). This obviously relates to the homogeneity of the results: only two depressed patients showed significant effect of incentive motivation on grip force. Since the vast majority of patients were unresponsive to monetary incentives, it is not surprising that no correlation was found with medications or clinical assessments. We also tested correlations between arousal effects and different clinical scores, with the hypothesis that they might relate to anxiety symptoms. We found no significant correlation (r = 0.20, r = −0.11, r = −0.10 for apathy, anxiety and depression respectively, all *P*>0.5).

## Discussion

Observing the behavior of depressed patients here showed that emotional arousal and incentive motivation are dissociable sources of effort production, in keeping with our previous demonstration using functional MRI in healthy subjects [21]. Precisely, we found two dissociations in depressed patients that were opposite to the patterns observed in healthy controls. First, emotional arousal did help patients to produce more force but potential rewards did not. Thus, the difficulty in energising behavior would not result from patients' emotional state, since negative emotions enhanced force production just as positive emotions did, but from a specific incentive motivation deficit. Second, while objectively patients did not produce more force, they subjectively felt having exerted more effort with higher monetary incentives. Thus, for depressed patients, their incentive motivation deficit would make seemingly easy tasks feel much harder.

The evidence found here for incentive motivation deficit shows that the process of translating potential rewards into behavioral activation is dysfunctional in major depression. A primary explanation could be that limbic basal ganglia circuits, which were shown to underpin incentive motivation effects in healthy subjects [19], [21], may be impaired in depressed patients. This would be consistent with the reduced ventral striatum response to rewards that has been reported in several studies [12], [13], [14]. It would also accord well with a similar incentive motivation deficit found in patients with a form of apathy (termed ‘auto-activation deficit’) induced by basal ganglia damage [20]. Another biological explanation may be dopamine dysfunction, since dopamine has been implicated in reward processing, response vigor and incentive motivation [26], [27], [28], [29]. Indeed, vulnerability to major depression has been linked to polymorphism in genes involved in dopamine metabolism and signaling [30]. In a previous study we showed that patients with Parkinson's disease (PD), who suffer from dopaminergic degeneration and often exhibit symptoms of apathy and depression, were still able to translate higher rewards into higher efforts, although to a lesser extent than healthy controls [20]. The difference found between PD and depressed patients' behavior shows that the incentive motivation deficit demonstrated here has some specificity and would not be observed in any hospitalized population. This difference also suggests that dopamine depletion alone may not be sufficient to explain the dramatic loss of incentive motivation observed in depressed patients. It should be noted however that the ventral striatum dopaminergic innervation is relatively preserved in PD [31], [32], which might account for the presence of incentive motivation effects. In any case, our results may support the use of dopaminergic medication in major depression, to restore the mechanisms that enable goals to energize behavior.

An alternative explanation to the incentive motivation deficit could be that depressed patients did not care about money, or missed the information about monetary payoff during the task. This explanation is ruled out by the fact that patients reported higher effort sensation following higher incentives. Thus, although patients did not show any effect of potential rewards in their motor responses, they took into account reward information in their performance judgments. Relative to controls, patients expressed opposite judgments: incentives made the task not easier but harder for them. A direct interpretation would be that patients do engage more effort, consistent with the finding that brain activations may be higher than in controls for a similar performance [33], but for some reasons this extra-effort fails to boost the motor command. A more sophisticated interpretation would be that the incentive motivation deficit is secondary to a distortion of performance judgments. Patients would guess that they are expected to give better performance but feel unable to fulfill this expectation, which would both increase fatigue sensation and prevent them to engage more effort.

In contrast with the absence of incentive motivation effects, emotional arousal helped patients to exert more effort. This dissociation might be surprising, since in most ecological situations, the two phenomena usually go together. Indeed, the sight of a prey or predator would trigger both emotional arousal, which activates the vegetative nervous system, and incentive motivation, which drives behavior towards goals. In this case, vegetative arousal may represent an adapted reaction that prepares the organism to fight or flee. However, we can theoretically conceive non-emotional goal-directed behavior, as when we take a shower (to get clean), and emotional arousal unrelated to goal-directed behavior, as when we watch a sad movie. This is the dissociation implemented in our experiment: emotional pictures incidentally modulated arousal levels without representing goals for the behavior, which remained winning money. As manifest in their high picture ratings, depressed patients were prone to emotional arousal, which facilitated effort production. This is consistent with studies reporting exacerbated response of the amygdala, anterior insula and ventrolateral prefrontal cortex in depressed patients [5], [6], [7]. On the contrary, healthy controls showed no significant effect of emotional arousal but a dramatic effect of incentive motivation. This could be interpreted as hyper-rational behavior, focusing on the goal and filtering out emotional distractors. It must be remembered however that the global analysis of variance showed no significant interaction between group and emotion factors, and that the younger half of healthy subjects did produce more force following arousing pictures, in replication of a previous study [21]. The most reliable difference between patients and controls therefore lies in the incentive motivation (not emotional arousal) processes that enable engaging more effort to obtain more reward.

We found no effect of emotional valence: positive and negative pictures equally increased force production. This seems at odds with the idea that depressed patients are more sensitive to negative stimuli, which was supported by many previous results [8], [9], [34], [35], [36], although not consistently [15], [37]. However, these studies used different measures, such as emotional judgment, mood state or brain activity. Exerted force is a specific measure that likely represents a consequence of vegetative arousal, thus a particularly reduced dimension of emotional reactions. Other dimensions of emotions and feelings may nonetheless be more pronounced for negative stimuli or memories in major depression. Vegetative arousal may be captured by other measures, such as skin conductance response. We measured skin conductance in some of our patients and found higher responses to both positive and negative pictures. Unfortunately, the data were not sufficient to conduct a proper statistical analysis. Also, we found that the arousal effect here was driven by social pictures, for both positive and negative valence. Thus, a limitation of our study is that we only used positive and non-social incentives (winning money) to suggest goals. It remains possible that negative incentives (potential punishments) would still activate avoidance behavior, and that social rewards would still activate approach behavior, in depressed patients.

In conclusion, our data bring strong empirical evidence for a specific incentive motivation deficit in depression. However, our measures of incentive motivation effects were not sufficiently sensitive to discriminate major depression at the individual level. At the group level, the task provided may serve as a useful tool to reveal an inability to energize behavior in order to attain goals. Such inability would explain why patients exhibit equally poor performance in tests assessing various motor and cognitive functions [38]. It is remarkable that almost all depressed patients failed to exert more effort to obtain more reward, despite the heterogeneity of individual histories. This means that the incentive motivation deficit is linked to the clinical picture (major depressive episode), and not to particular etiologies or treatments. We cannot formally conclude that the motivation deficit is a state (not a trait) effect however, as we have not tested patients after recovery from their depressive episode. Since the motivation deficit was present in almost all patients, we could not find any relation with treatments nor with clinical features. More precisely, the absence of motivation effects was observed whatever the type of medication and whatever the severity of depression, anxiety and apathy scores on clinical scales. Correlations might yet be found assessing a larger sample of less severe patients, who would exhibit more variability. Also, patients were tested here before the expected therapeutic benefit from their antidepressant medication. Further studies will examine whether our incentive force task could be used to predict the clinical outcome of the various anti-depressant treatments.
